# MedsOnCall Pager App: A Pilot Project for Practicing Safe Clinical Decision-making

**DOI:** 10.7759/cureus.3205

**Published:** 2018-08-27

**Authors:** Nada Gawad, Heather McDonald, Isabelle Raiche, Fraser Rubens

**Affiliations:** 1 Surgery, University of Ottawa, Ottawa, CAN; 2 Surgery, University of Ottawa, ottawa, CAN; 3 Surgery, The Ottawa Hospital, Ottawa, CAN; 4 Surgery, Ottawa Heart Institute, ottawa, CAN

**Keywords:** simulation program, medical student education, resident education, mobile apps, clinical decision-making

## Abstract

Errors in clinical decision-making contribute to approximately half of in-hospital adverse events. The steep learning curve when students transition to residents is particularly susceptible to increased errors. Decision-making skills are a major contributor to preparedness for residency and educators agree that decision-making should be purposefully taught and tested. Despite this, little structured assessment of decision-making currently exists. This innovation report describes the development and piloting of the MedsOnCall (MOC) Pager App, a simulated pager program designed as a learning and assessment tool for senior medical students and junior residents to practice safe clinical decision-making as they transition between these two roles. Learners are randomly “paged” by the app about a list of virtual patients. To answer, they must integrate pertinent patient information efficiently. Learners then receive a page-management question that further probes their decision-making skills by asking them to consider the urgency and their level of confidence when determining the virtual patient’s needs. The pilot version of the app was successfully alpha-tested in 2016 and 2017 with twenty fourth year medical students at our institution. Subjectively, students greatly enjoyed using the MOC Pager app to practice answering pages in a safe environment. The app was then adapted for the National Cardiac Surgery Bootcamp in 2017 for use by first-year residents. With demonstrated success as a pilot project, our group aims to rebuild the app for customizable use by multidisciplinary learners anywhere in the world simultaneously. We also plan to collect validity evidence, integrate in-app feedback capability, and disseminate the app on multiple platforms.

## Introduction

An estimated 98,000 people die each year due to medical errors [1]. Of the in-hospital errors, approximately half involve errors in clinical decision-making [2]. Most of the research on errors has focused on teaching hospitals, where residents provide much of the direct patient care [3]. The impact of learners is especially apparent during the steep learning curve in transitioning from medical student to resident [4], when the responsibility of making a final decision is thrust upon the learner. These decisions have consequences on the outcomes of real patients. This is demonstrated by the “July Effect” where increased errors leading to patient mortality occurs with the influx of new residents [4].

Accordingly, decision-making skills majorly contribute to residency preparedness and educators agree that decision-making should be purposefully taught and tested by medical schools, licensing bodies, and specialty societies [5]. Despite the focus on including decision-making as part of establishing competence, little structured assessment of competent decision-making currently exists in undergraduate or postgraduate medical education.

Assessment in medical education has increasingly relied on simulation due to time and resource constraints, as well as an emphasis on patient safety [6,7]. While posing no risk to real patients, simulation-based medical education forces trainees to act and respond as they would in a real situation, thus enhancing learning through contextually-relevant experience [8]. Mobile apps have become increasingly within medical education due to their portability, easy access, and enhancement of medical learning in the clinical setting [9], and thus provide the ideal platform for simulation-based learning. The purpose of this innovation report is to describe the conception and development of the MedsOnCall (MOC) Pager App, a simulated pager program designed to purposefully assess clinical decision-making skills.

## Technical report

Approach

The MOC Pager App is the first known simulated pager app designed to be used as a learning and assessment tool for learners to practice safe clinical decision-making. Our group pioneered this unique approach to the learning and assessment of decision-making skills after an informal needs assessment at our institution suggested that graduating medical students were lacking the practical decision-making skills required for the management of patients on the floor. Clinical scenarios repeatedly identified as challenging for these students included common issues such as fevers, tachycardia, anticoagulation, and fluid resuscitation. The inability or insecurity in managing such common scenarios is concerning given that first-year residents may independently manage patients on the floor, specifically when they are designated as first-call for overnight pages. Beyond knowing how to answer the page, residents must be aware of their limitations with respect to managing each page on their own and with what degree of urgency they must respond to the page [10].

**Figure 1:**
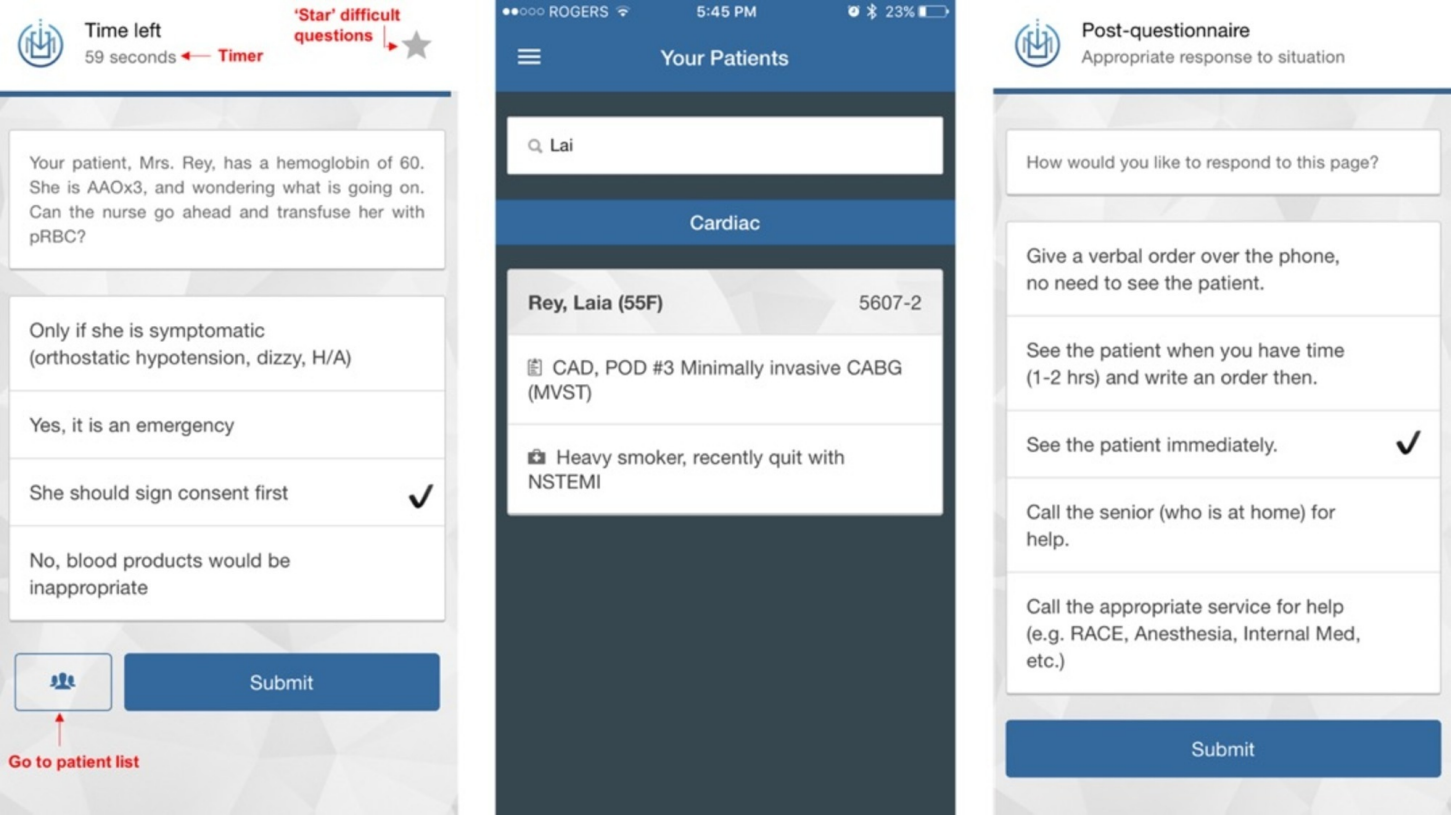
Screenshots of a sample page, patient information, and page-management question from the MedsOnCall (MOC) Pager App. Features within the app, such as a timer, the ability to star difficult questions, and the ability to easily navigate from the question to the patient list are shown in red text.

Based on a list of virtual patients, learners using the MOC Pager App as a clinical adjunct are randomly “paged” by the app. To answer the page correctly, they must consider the patient’s pertinent information as displayed on the virtual patient list, thus requiring the learner to integrate their knowledge and take ownership of these patients. The app provides the learners with choices of possible actions, and the learner must choose the most appropriate answer within a given period of time to simulate the efficiency and triaging required of patient management on the wards. After each page, learners receive a page-management question that further probes their decision-making skills by asking how they would respond to the page; i.e., give orders over the phone, see the patient in an hour, see the patient immediately, call someone more senior for help, or consult another specialty. The page management question forces the learner to consider their medical knowledge, confidence, the patient’s safety, and when to ask for help. Screenshots of the page, patient information, and page-management question are shown in Figure 1.

The pages are sent to learners by the app in a randomized order at unknown times throughout the day, and must be opened before the next page arrives. This is orchestrated to simulate the need for efficiency and multi-tasking on the ward. Once each page is opened, the learner has only two minutes to answer the page to simulate the need to make time-sensitive clinical decisions and challenge their ability to efficiently use other resources for help.

Unlike many other medical education apps, the MOC Pager is not a database app where information can be searched, but rather a means of knowledge and clinical decision-making practice and assessment. In this regard, the MOC Pager is a form of portable simulation-based learning. The MOC Pager simulates the random appearance, high volume, and varied urgency of pages received on the wards.

Incorporation of the MOC Pager app into existing undergraduate and/or postgraduate curricula allows for the purposeful assessment of decision-making and knowledge in the clinical setting as students become residents. As such, the intended learners include both senior medical students and junior residents, with the purpose of easing the large jump when transitioning between these two roles. Most traditional methods of assessment do not purposefully assess decision-making skills, and thus the app’s main goals are to provide programs with quantitative data on the decision-making skills of learners as well as to improve teaching around decision-making and time management skills. Furthermore, unlike rotation-based assessment, errors do not result in harm to patients since the app simulates patient care. And unlike written tests, assessment is contextually authentic and performed in real-time in the clinical setting where multiple factors relating to the patient and time constraints must be considered. As such, the MOC Pager mimics the need for multi-tasking and efficiency.

Outcomes

The MOC Pager app is currently available for download as a free iOS app in the App Store. Members of our group conceived and/or designed the app in 2015, and alpha-testing of the pilot app was launched in 2016 as the assessment tool used in the one-week Perioperative Management elective at the University of Ottawa designed by our group [11]. Participants were twenty fourth year medical students transitioning to residency in either surgical or anesthesia specialties. The pilot version is based on a list of 30 virtual surgical patients, with information such as their admitting service, scheduled surgery, and past medical history. Each student received approximately 80 pages over the course of the one-week elective.

A 2017 study quantified students’ progress during the aforementioned Perioperative Management elective using the MOC Pager App. As described by McDonald et al. [11], a mixed-effects model of 1,329 pages answered by 20 students (five students in 2016, 15 students in 2017) demonstrated a significant increase in the proportion of correctly-answered pages over the course of the elective week (p = 0.04). Subjectively, students felt more comfortable managing patients and answering pages (p < 0.001) and reported greatly enjoying the MOC Pager app to practice answering pages in a safe environment.

On exit surveys administered to the 20 participants (100% completion rate) during programmatic evaluation, students cited the MOC Pager App as “an excellent learning tool” and an “innovative method of assessment”. There were no negative comments toward the app. Over the one-week elective, participating students specifically described improvements such as in “determining priorities”, “triaging”, “understanding concern(s)”, “calling for help”, and “knowing which patient to see first when there are sick patients”, although it is unknown to what degree these comments pertain to the MOC Pager App and/or the elective curriculum content.

In terms of areas of improvement described on the exit surveys, students cited issues with technical glitches in the app and that the original time period of one minute to answer each question was insufficient. Because of this feedback, bugs were fixed and the length of time to answer each question was increased to two minutes. These changes were implemented in the 2017 pilot of the app in the same elective.

Most recently, the app was modified for use in the National R1 Cardiac Surgery Bootcamp in Ottawa in 2017. Question developers for the Cardiac Surgery Bootcamp reported easily constructing new specialty-specific questions and patients based on the existing template. Currently, however, the app can only be used by one program at a time, thus limiting its expansion to other programs and institutions.

## Discussion

This innovation report describes the development and piloting of the MOC Pager App, which serves as a unique adjunct to clinical training and addresses the currently unmet need of practicing and assessing clinical decision-making as a means of improving patient safety. Although clinical decision-making can be assessed in the clinical environment, current barriers include the required supervision and risk to patients. The simulation-based assessment offered by the MOC Pager App resolves these obstacles by facilitating independent use by learners, providing training programs with collated and numerical feedback, and ensuring no harm to patients during practice. Preliminary results published from a study on a Perioperative Management elective, in which the app was piloted, demonstrated promising and overall positive quantitative and subjective results, while providing direction with respect to areas of improvement [11]. These preliminary results, along with the current limitations of the app, have prompted rebuilding the app to enable further development and expansion prior to beta-testing.

The most limiting feature of the pilot app is that in its current form, the MOC Pager can only be used by learners in one program at a time and cannot be easily adapted to multiple specialties. Our group aims to rebuild the MOC Pager App to consist of multiple user interfaces (student and teacher/administrator). This will allow different programs and institutions to easily customize the app’s content to their desired audience. When students log on to the app, they will be able to register to their program and thus focus their clinical decision-making practice on their field of study. In addition, depending on the needs of each program, the app will be programmable to either provide immediate feedback to the learner or to withhold the answers until later classroom-based discussion. In either option, the rationale provided would be based on answers deemed best by a panel of experts, therefore providing a more complete formative assessment strategy. Furthermore, the rebuilt app would be able to be used on several platforms (iOS, Android, desktop) thus allowing to be used at any institution in the world.

With respect to evaluating the MOC Pager App as a decision-making assessment tool, validity evidence to support the results generated by the app needs to be collected. The appropriateness of the interpretation of data provided by the app and the inferences made from test scores must be determined by collecting the five sources of validity evidence. Specifically, test content and response process validity evidence should be gathered during test development, and internal structure, relations to other variables, and consequences of testing should be determined during and after administration of the MOC Pager App [12].

## Conclusions

With presentation of our preliminary results, we have garnered the interest of multiple programs who have agreed to participate in beta-testing the rebuilt app. We intend to work with these programs to create their own virtual patient list and database of questions such that learners in any discipline, at any level of training, at any institution can download the app and participate in their own program’s version simultaneously. Validity evidence will be collected in the beta-testing phase. After beta-testing, collecting validity evidence, and refining the rebuilt MOC Pager App, we plan to disseminate the app to improve the learning and assessment of clinical decision-making as medical students transition into residents. It is our hope that this innovative approach to practicing safe clinical decision-making will improve patient safety through simulation-based learning and assessment.
